# The prevalence of non-contact muscle injuries of the lower limb in professional soccer players who perform Salah regularly: a retrospective cohort study

**DOI:** 10.1186/s13018-020-01955-5

**Published:** 2020-09-24

**Authors:** Eduard Bezuglov, Oleg Talibov, Mikhail Butovskiy, Anastasiya Lyubushkina, Vladimir Khaitin, Artemii Lazarev, Evgeny Achkasov, Zbigniew Waśkiewicz, Thomas Rosemann, Pantelis T. Nikolaidis, Beat Knechtle, Nicola Maffulli

**Affiliations:** 1 Sechenov First Moscow State Medical University (Sechenov University), Moscow, Russian Federation; 2 Federal Research and Clinical Center of Sports Medicine and Rehabilitation of Federal Medical Biological Agency, Moscow, Russian Federation; 3 High Performance Sports Laboratory, Moscow Witte University, Moscow, Russian Federation; 4 Moscow State University of Medicine and Dentistry, Moscow, Russian Federation; 5 FC Spartak, Moscow, Russian Federation; 6 “Smart Recovery” Clinic, Moscow, Russian Federation; 7 FC Zenit Saint Petersburg, Saint Petersburg, Russian Federation; 8 Institute of Sport Science, Jerzy Kukuczka Academy of Physical Education, Katowice, Poland; 9 Institute of Primary Care, University of Zurich, Zurich, Switzerland; 10 Exercise Physiology Laboratory, Nikaia, Greece; 11 Medbase St. Gallen Am Vadianplatz, St. Gallen, Switzerland; 12 Department of Musculoskeletal Disorders, Faculty of Medicine, Surgery and Dentistry, University of Salerno, Via S. Allende, 84081 Baronissi, SA Italy; 13 Centre for Sports and Exercise Medicine, Barts and The London School of Medicine and Dentistry, Mile End Hospital, Queen Mary University of London, 275 Bancroft Road, London, E1 4DG England; 14 School of Pharmacy and Bioengineering, Keele University Faculty of Medicine, Thornburrow Drive, Stoke-on-Trent, England

**Keywords:** Hamstring, Muscle injury, Soccer, Prevention, Hamstring injuries

## Abstract

**Background:**

The present study assessed the prevalence of non-contact muscle injuries of the lower limbs, including hamstring injuries, in professional Russian soccer players who regularly perform Salah, an obligatory Muslim prayer performed 5 times a day.

**Methods:**

Using a retrospective cohort study design, 68 professional male soccer players (excluding goalkeepers), 34 of whom were Muslims regularly performing Salah (exposure group) and 34 were randomly chosen non-Muslim players (control group), were included in the study. The groups were similar in their playing leagues, field positions, age (27 ± 3.1 vs 28 ± 4.2 years), and body mass index (22 ± 1.2 vs 23 ± 0.92 kg/m^2^).

**Results:**

The incidence of hamstring injury was significantly lower in the exposure group (2 vs 14, *p* = 0.0085). A declining trend for the number of muscle injuries (either hamstring or not) was observed in the exposure group (11 vs 27, *p* = 0.0562). Two players in the exposure group and 11 in the control group (*p* = 0.0115, OR 0.1307, 95% CI 0.0276 to 0.5698) suffered a hamstring injury, with no statistically significant difference in the occurrence of other injuries. The total amount of the training and play days missed because of hamstring and other muscle injuries was significantly lower in the exposure group (24 vs 213 days, *p* = 0.0043, and 200 vs 344 days, *p* = 0.0066, respectively).

**Conclusion:**

The prevalence of non-contact muscle injuries, including hamstring injuries, was lower in professional Russian soccer players who regularly performed Salah.

## Background

Thirty-one to 41% of all injuries in soccer involve the muscles, with most occurring in the thigh [1–3]. The hamstring muscles (comprising the biceps femoris, semimembranosus, and semitendinosus muscles) account for up to 37% of all muscle injuries in soccer players, a number on the increase. The second and third most prevalent injuries are the injuries of the adductor muscles of the hip and the quadriceps femoris muscle (23% and 19%, respectively) [4, 5]. During a competitive season, a professional European soccer team is expected to experience approximately 15 muscle injuries, 4–6 of which will affect the hamstrings. Although often of little clinical relevance, these injuries do impact negatively on athletes, who on average are not able to play in 3–4 games and require about 14 days to return to sport [2, 6]. In addition, recurrence of muscle injuries is common, with a prevalence from 16 to 24% [7, 8].

Age, previous history of injuries, imbalance between strength and flexibility, and decrease in both eccentric power and mobility all play an important role in non-contact muscle injury [9]. Eccentric exercises aimed at hamstrings are currently considered the best method to prevent their injury [10, 11]. Most often, muscle injuries occur during eccentric contraction [12, 13], and eccentric exercises should be included in training programs to prevent muscle injury. Some studies highlighted the association between lumbar and pelvic mobility and the frequency of hamstring injury [14].

Considering the high prevalence of injuries among professional soccer players, injury prevention is a pressing problem in sports medicine, and specific programs have been developed for this purpose [11]. In addition, there is great interest in predicting hamstring injury [15, 16].

Salah (also called Salat and Namaz) is a traditional Muslim prayer. In traditional Islam, Salah is performed five times a day. Each Salah consists of a set of repeated movements called Rakats. Up to 48 Rakats may be performed daily, and at least 17 of them are mandatory. Rakat consists of a specific sequence of 7 to 9 postures [17]. Compulsory Salah (so-called Fard) includes 5 sets of prayer movement sequences: Fajr (the dawn prayers—2 sets), Zuhr (the afternoon prayer—4 sets), Asr (the late afternoon prayer—4 sets), Maghrib (the evening prayer—3 sets), and Isha (the night prayer—4 sets). Overall, a Sunni Muslim should repeat the prayer movements at least 17 times every day [18]. Therefore, the overall number of postures taken when performing Salah cannot be less than 119 per day [19]. Each of the nine postures has a specific duration, which varies from 3–4 to 40–60 s.

The specific posture sequence and duration are as follows: Takbir (standing, 3–4 s), Qayyam (standing, 40–60 s), Ruku (bowing, 10–12 s), Qayyam (standing, 5–6 s), Sajdah (prostration, 10–12 s), Jalsа (sitting, 6–8 s), Sajdah (prostration, 10–12 s), Jalsa (sitting, 40–60 s), and Salam (sitting with head turns to the right and to the left, 3–4 s).

The regular practice of Salah may positively impact an individual’s health, including the health of the musculoskeletal, cardiovascular, and nervous systems [14, 20–22].

**Fig. 1:**
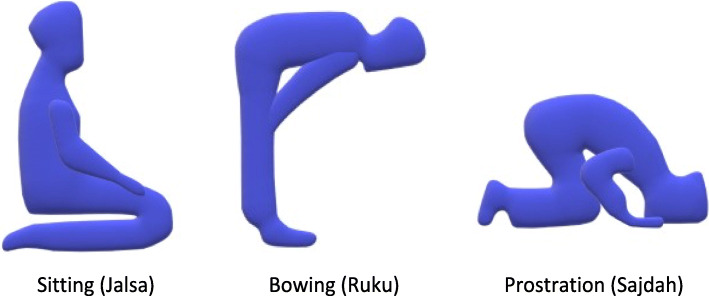
Jalsa, Ruku, and Sajdah postures

Jalsa, Ruku, and Sajdah postures involve all the muscles of the lower limb and the lumbosacral spine, as well as all the large joints (Fig. 1). Most of the movements in these postures involve eccentric loading of certain muscles of the lower limbs. The total time spent in these postures during the day is at least about 20 min.

Ruku (bowing) posture strengthens the back and increases hip mobility and the mobility of the popliteus tendon. It reduces spinal, back, and neck stiffness and helps to improve body posture, balance, and coordination. Performing movements similar to Ruku positively affects the lower spine and body stability [23].

Jalsa (sitting) posture leads to the extension of the muscles of the shins and buttocks and to the maximum flexion of the knee joint [24]. This and similar postures reinforce the core and muscles and the muscles of the lower back, an important factor in preventing the development of pain in this region [25]. The regular performance of Salah by soccer players may affect their rate of muscle injuries.

The goal of this study was to assess the prevalence of non-contact muscle trauma in professional soccer players who regularly perform Salah. We wished to test the null hypothesis of no difference in the rate of prevalence of lower limb muscle injuries in players who performed Salah and players who did not.

## Methods

The study was approved by the local Ethics Committee of Sechenov First Moscow State Medical University with the number N 11-19. This retrospective study involved two cohorts of participants. Professional male soccer players from the two major Russian soccer leagues (the Russian Premier League (RPL) and the Football National League (FNL)) were included. The exposure group included 34 Muslim players who regularly performed the five daily prayers, with a minimum of 17 Rakats every day. The control group included 34 randomly selected non-Muslim soccer players from the same soccer teams. Goalkeepers were not included in the study given the requirements of their playing position and training methods. Given the objectives of the study, the commitment to Salah, and not religion in and by itself, was selected as the inclusion criterion.

Data on non-contact muscle injuries suffered by players during the season were selected as the primary outcome. The season lasted from July 28, 2018, to May 26, 2019, for the RPL and from July 17, 2018, to May 25, 2019, for the FNL. The data on the characteristics of injuries studied in the present investigation included the presence of non-contact injuries of the hamstring muscles, other non-contact muscle injuries of the lower extremities, and all other injuries of the lower limbs (both contact and non-contact), as well as the duration before returning to regular training activity after suffering non-contact muscle injuries.

Statistical analysis was performed using the GraphPad Prism application version 8.0.0 for Mac OS X. No imputation or substitution of missing values was performed. The normality of the collected quantitative data (i.e., age and BMI) was tested using the Kolmogorov-Smirnov test. Normally distributed data were described using mean (M), standard deviation (SD), and min-max ranges. For other distributions, median (Me), interquartile (Q1–Q3), and min-max ranges were used.

A two-sample independent *t* test with Welch’s correction for unequal variances was used to assess the intergroup differences in case of normal distribution. The Mann-Whitney *U* test was used to assess the significance of intergroup differences for non-normal distribution. The difference between means with standard deviation and 95% confidential intervals of the difference between medians was given.

Categorical data (i.e., player position, league affiliation, outcomes) were described using frequency charts showing an absolute value and its percentage share. Fisher’s two-tailed exact test was used to assess the intergroup differences. The odds ratios (OR) are provided along with the 95% confidence intervals (95% CI) calculated using the Baptista-Pike method. The chi-squared test was used to test the differences in 3 × 2 contingency tables. Values at *p* < 0.05 were considered statistically significant. The total number of injuries and the number of missed training or play days were recorded for each of the groups studied. The Mann-Whitney *U* test was used to test the significance of the observed difference.

## Results

The descriptive summary of the subjects of the study is presented in Table 1. There were no differences in age (difference between means is 0.2941 ± 0.891; *p* = 0.7426), BMI (difference between means is 0.1462 ± 0.254; *p* = 0.5670), total games played (95% of median difference − 2 to 5; *p* = 0.2857), played minutes (95% of median difference − 22 to 618, *p* = 0.0722), player position (*p* = 0.2043), and league affiliation (*p* > 0.9999) between the players of the two groups.

**Table 1:** Overview of the characteristics of the subjects participating in the study

	Salah group (*n* = 34)	Control group (*n* = 34)	*p* value
**Age (M ± SD; min–max)**	27 ± 3.1; 21–32	28 ± 4.2; 20–34	0.7426
**BMI (M ± SD; min–max)**	22 ± 1.2; 20–25	23 ± 0.92; 21–24	0.5670
**Games (Me; Q1–Q3; min–max)**	23; 17–27; 10–36	24; 22–27; 10–36	0.2857
**Play minutes (Me; Q1–Q3; min–max)**	1528; 987–2020; 705–2876	2033; 1444–2350; 827–3195	0.0722
**Player position**			
**Defender,** ***n*** **(%)**	9 (26)	16 (47)	0.2043^#^
**Midfield,** ***n*** **(%)**	19 (56)	13 (38)	
**Forward,** ***n*** **(%)**	6 (18)	5 (15)	
**League**			
**Russian Premier League,** ***n*** **(%)**	24 (71)	23 (68)	> 0.9999^##^
**Football National League,** ***n*** **(%)**	10 (29)	11 (32)	

^#^Chi-square test

^##^Exact Fisher’s test

The incidence of hamstring injury was significantly lower in the Salah group (2 vs 14, *p* = 0.0085) (Table 2). A declining trend for the number of muscle injuries’ general amount (either hamstring or not) was observed in the Salah group (11 vs 27, *p* = 0.0562).

**Table 2:** The total number of injuries in both groups

	Salah group (*n* = 34)	Control group (*n* = 34)	*p* value
Hamstring injuries (*n*)	2	14	**0.0085**
General muscle injuries (*n*)	11	27	0.0562
Non-muscle injuries (*n*)	16	12	0.4879
All traumas (*n*)	27	39	0.3234

The number of players who received one, two, or three and more non-contact muscle injuries was calculated for both groups and is presented in Table 3.

**Table 3:** The number of injured players

	Salah group (*n* = 34)	Control group (*n* = 34)	*p* value
Total injuries			
Total injured players, *n* (%)	21 (62)	20 (59)	> 0.9999
One injury, *n* (%)	16 (47)	7 (21)	**0.0392**
Two injuries, *n* (%)	4 (12)	8 (24)	0.3405
Three or more injuries, *n* (%)	1 (3)	5 (15)	0.1974
Hamstring muscle injuries			
Total injured players, *n* (%)	2 (6)	11 (32)	**0.0115**
One injury, *n* (%)	3 (9)	9 (26)	0.1092
Two injuries, *n* (%)	0	1 (3)	> 0.9999
Three or more injuries, *n* (%)	0	1 (3)	> 0.9999
Other muscle injuries			
Total injured players, *n* (%)	11 (32)	17 (44)	0.2177
One injury, *n* (%)	11 (32)	9 (26)	0.7906
Two injuries, *n* (%)	0	5 (15)	0.0534
Three or more injuries, *n* (%)	0	2 (6)	0.4925
Non-muscle injuries			
Total injured players, *n* (%)	13 (38)	10 (29)	0.6088
One injury, *n* (%)	11 (32)	8 (24)	0.5896
Two injuries, *n* (%)	1 (3)	2 (6)	> 0.9999
Three or more injuries, *n* (%)	1 (3)	0	> 0.9999

The total number of injured players with hamstring trauma was significantly lower in the Salah group (2 vs 11, *p* = 0.0115; OR 0.1307, 95% CI 0.0276 to 0.5698).

There was no statistically significant difference in the total number of injuries (including non-muscle injuries) between the observed groups.

The data on the total number of missed training and play days caused by injuries is presented in Table 4.

**Table 4:** The total number of training and play days missed due to injury

	Salah group (*n* = 34)	Control group (*n* = 34)	*p* value
Hamstring injuries (*n*)	24	213	0.0043
General muscle injuries (*n*)	200	344	0.0066
Non-muscle injuries (*n*)	148	335	0.7469
All traumas (*n*)^a^	348	679	0.4285

^a^Not equal to the sum of three preceding rows as combined injuries were observed in several cases.

The Salah group players also missed less training and play days due to injury of the hamstring or other muscles (see Table 4). The observed total time difference was 24 vs 213 days for hamstring injuries (*p* = 0.0043) and 200 vs 344 (*p* = 0.0066) for the general number of muscle injuries.

## Discussion

The main finding of this investigation is that the prevalence of non-contact muscle injuries, including those to the hamstring muscles, is lower in soccer players who regularly perform Salah.

Although the reasons for such finding is likely to be multifactorial, we hypothesize that the eccentric lengthening of the muscles of the lower limbs during certain movements undertaken several times per day during prayers plays a major role. The positive effect of performing Salah on the muscles of the lower limbs has been shown in previous studies, but such investigations were not conducted on athletes [26, 27].

Various injury prevention programs in soccer players have produced a reduction in both the overall injury rate and the hamstring injury rate [17, 28, 29].

Salah, a religious practice, includes a set of movements in a codified sequence. If performed daily, they can positively affect the joint mobility in the knee, hip, and ankle joints, as well as the lumbar and cervical spine [12]. The movements performed during Salah are similar to the eccentric exercises aimed at the hamstrings [30, 31], as well as with the classic hatha yoga postures (“asanas”). However, in general, the movements performed during Salah are easier to perform and do not require special skills and are undertaken by the players independent from their training regimen [17].

We are aware that the prevalence of non-contact muscle injuries depends not just on performing specific exercises, but also on the age, number of days between matches, and history of previous injuries [30, 32]. One of the factors that may influence the prevalence of muscle injury may be alcohol consumption, which is likely to be significantly lower in Muslim football players than in the control group [33–35].

On the negative side, one of the prayers is performed at night and the other before sunrise. This can negatively affect the quality and duration of sleep and be a negative factor for physical performance and injury occurrence [36, 37]. In the present study, the possible effects of sleep quality, diet, and alcohol consumption were not assessed. Future studies will be needed to clarify these issues.

Several techniques have been proposed for treating hamstring injury. Some authors proposed the use of platelet-rich plasma [38, 39] or surgical treatment [40], but depending on the degree of damage, the treatment is quite time-consuming.

We are aware that this is not a randomized study. For religious reasons, Muslim players would not be able to participate in a study where they were asked not to be allowed to pray, and non-Muslim players may not give consent to undertake exercise late at night and before dawn. In addition, the randomized approach to the selection of the control group for the observational study may contain flaws that bias the final estimate. Prospective cohort studies are the preferred design for observational studies in soccer players as per the guidelines of the Union of European Football Associations [7]. Potential confounders, including total time played in matches and spent during practice as well as field position etc., are a further limitation of the present study. The retrospective nature of the study and the mode of data collection do not allow to determine some important features of muscle injuries, such as time to first trauma and hazard rate. Therefore, the results of this study should be regarded as preliminary and interpreted with caution. Nevertheless, the data collated seem compelling and may be used to inform larger prospective investigations.

## Conclusions

The prevalence of non-contact muscle injuries, including hamstring injuries, was lower in professional Russian soccer players who regularly performed Salah. Larger prospective investigations are required to confirm the data obtained in this retrospective study.

## Data Availability

The datasets used and/or analyzed during the current study are available from the corresponding author on reasonable request.

## Abbreviations

RPL: Russian Premier League
FNL: Football National League
BMI: Body mass index
OR: Odds ration
CI: Confidence interval
